# The High Calcium, High Phosphorus Rescue Diet Is Not Suitable to Prevent Secondary Hyperparathyroidism in Vitamin D Receptor Deficient Mice

**DOI:** 10.3389/fphys.2017.00212

**Published:** 2017-04-10

**Authors:** Sarah M. Grundmann, Corinna Brandsch, Daniela Rottstädt, Hagen Kühne, Gabriele I. Stangl

**Affiliations:** ^1^Institute of Agricultural and Nutritional Sciences, Martin Luther University Halle-WittenbergHalle, Germany; ^2^Competence Cluster for Nutrition and Cardiovascular Health (nutriCARD), Halle-Jena-LeipzigHalle, Germany

**Keywords:** vitamin D receptor, rescue diet, calcium, phosphate, parathyroid hormone, fibroblast growth factor 23, mice

## Abstract

The vitamin D receptor (VDR) knockout (KO) mouse is a common model to unravel novel metabolic functions of vitamin D. It is recommended to feed these mice a high calcium (2%), high phosphorus (1.25%) diet, termed rescue diet (RD) to prevent hypocalcaemia and secondary hyperparathyroidism. First, we characterized the individual response of VDR KO mice to feeding a RD and found that the RD was not capable of normalizing the parathyroid hormone (PTH) concentrations in each VDR KO mouse. In a second study, we aimed to study whether RD with additional 1 and 2% calcium (in total 3 and 4% of the diet) is able to prevent secondary hyperparathyroidism in the VDR KO mice. Wild type (WT) mice and VDR KO mice that received a normal calcium and phosphorus diet (ND) served as controls. Data demonstrated that the RD was no more efficient than the ND in normalizing PTH levels. An excessive dietary calcium concentration of 4% was required to reduce serum PTH concentrations in the VDR KO mice to PTH levels measured in WT mice. This diet, however, resulted in higher concentrations of circulating intact fibroblast growth factor 23 (iFGF23). To conclude, the commonly used RD is not suitable to normalize the serum PTH in VDR KO mice. Extremely high dietary calcium concentrations are necessary to prevent secondary hyperthyroidism in these mice, with the consequence that iFGF23 concentrations are being raised. Considering that PTH and iFGF23 exert numerous VDR independent effects, data obtained from VDR KO mice cannot be attributed solely to vitamin D.

## Introduction

The vitamin D receptor (VDR) knockout (KO) mouse is an often used model to study the role of vitamin D in pathophysiological processes and for disease prevention. However, VDR KO mice are hypocalcaemic and characterized by a secondary hyperparathyroidism and a rickets phenotype (Li et al., 1997; Song et al., 2003). To combat these attendant symptoms and biochemical changes, it is recommended to feed the VDR KO mice a high calcium, high phosphorus diet. This type of diet is termed rescue diet (RD) and was first developed by Kollenkirchen et al. (1991). The classical RD contains 2% calcium and 1.25% phosphorus, and is considered to be able to normalize serum levels of calcium and parathyroid hormone (PTH) and to prevent rickets in these mice (Li et al., 1998). This type of diet is used in nearly all studies conducted with VDR KO mice. Currently, three different strains of VDR KO mice, which have been reported by Van Cromphaut et al. (Leuven strain) (van Cromphaut et al., 2001), Yoshizawa et al. (Tokyo strain) (Yoshizawa et al., 1997), and Li et al. (Boston strain) (Li et al., 1997), are available. These strains show comparable degrees of hyperthyroidism when fed a diet with normal calcium and phosphorus contents. The PTH increase in the VDR KO mouse models placed on such a normal diet is on average 25 times higher than that observed in the wild type (WT) mice fed the same diet (Li et al., 1997; van Cromphaut et al., 2001; Song et al., 2003). Independent of the VDR KO mouse strain, some data indicated that it is possible to prevent secondary hyperparathyroidism by feeding the RD (Li et al., 1998; Kaneko et al., 2011), while other and own data showed that VDR KO mice fed the RD had extremely high concentrations of circulating PTH when compared with WT mice (Song et al., 2003; Shiizaki et al., 2009; Kühne et al., 2016). Song et al. (2003), who used the Tokyo strain, described that the RD in comparison to a normal diet was not capable of reducing the PTH concentrations in VDR KO mice. Data presented by Shiizaki et al. (2009) showed obviously higher PTH concentrations in VDR KO mice (also Tokyo strain) fed the RD than in the WT mice. Own data obtained from VDR KO mice (Boston strain) demonstrated marked differences in the individual response of VDR KO mice to the RD (Kühne et al., 2016). These data raised doubts as to whether the RD is an appropriate diet to normalize, in particular, the PTH serum concentrations in each VDR KO mouse. Despite these findings, most published data of VDR KO mouse studies did not provide any information on circulating concentrations of PTH (Rummens et al., 2003; Simpson et al., 2007; Chen et al., 2015; Sakai et al., 2015).

Vitamin D Receptor (VDR) Knockout (KO) mice that suffer from a secondary hyperparathyroidism show a series of metabolic disturbances, making it impossible to distinguish between causal effects of VDR and secondary effects caused by pathological PTH concentrations. Thus, any conclusion drawn from VDR KO mouse studies might be derived from a rather heterogeneous group of mice that may differ in their mineral status and PTH response. This prompted us to characterize the individual responses of VDR KO mice to the RD, considering age and sex of the animals, and to define the dietary calcium concentration which is capable of normalizing the circulating PTH concentrations in each VDR KO mouse. Because dietary calcium is a potent stimulator of the fibroblast growth factor 23 (FGF23) (Rodriguez-Ortiz et al., 2012; David et al., 2013), which regulates both vitamin D and phosphate metabolism (Shimada et al., 2005), we also analyzed serum concentrations of intact FGF23 (iFGF23) in all mice.

## Materials and methods

### Animals and study design

All mice included in our investigations were housed in pairs in a room controlled for temperature (22 ± 2°C), light (12-h light, 12-h dark cycle) and relative humidity (50–60%). The experimental procedures described below followed the established guidelines for the care and handling of laboratory animals according to the National Research Council (US) Committee for the Update of the Guide for the Care and Use of Laboratory Animals (2011) and were approved by the local government (Landesverwaltungsamt Sachsen-Anhalt, Germany, approval number: H1-4/T1-14). Homozygous VDR KO mice obtained by mating heterozygous B6.129S4-*Vdr*^*t*^^m1Mbd^/J mice (Boston strain, Jackson Laboratories, Bar Harbor, USA) and corresponding homozygous WT mice were used for the studies.

In the first study, we characterized the response of VDR KO mice to feeding a commercial RD containing 2% calcium and 1.25% phosphorus (S8852-S010, ssniff Spezialdiäten GmbH, Soest, Germany) by analyzing serum concentrations of calcium, inorganic phosphate, PTH and iFGF23 and compared them with those of WT mice fed the same diet. Both genotype groups consisted of males and females and were fed the RD from weaning to 6 and 13 months of age, respectively. Each genotype-, age- and sex-specific group comprised 5 to 6 mice.

In the second study, 2-month-old male VDR KO mice were fed a semi-synthetic basal diet [20% casein, 20% sucrose, 20% lactose, 13% starch, 5% cellulose, 7% soybean oil, and 2% vitamin and mineral mixture according to AIN-93G (Reeves et al., 1993)] that differed in the mineral concentrations. From weaning to the age of 2 months all VDR KO mice received the commercial RD to reduce the risk of osteomalacia during the trial period. At the start of the trial group 1 of the VDR KO mice received the basal diet with 0.3% calcium and 0.3% phosphorus, termed normal diet (ND) according to the AIN recommendations (Reeves et al., 1993). The same diet was fed to WT mice which were used as reference group to specify normal mineral and PTH concentrations. Group 2 of the VDR KO mice were fed the basal diet, supplemented with 2% calcium and 1.25% phosphorus (RD). Groups 3 and 4 of VDR KO mice received the RD which was supplemented with 1 and 2% of additional calcium (RD+1 and RD+2, respectively). Calcium and phosphorus were added to the diet as calcium carbonate and dicalcium phosphate. Each group comprised 5 to 6 mice. The experimental diets were fed for 8 weeks to create experimental conditions comparable to previous VDR KO mouse studies.

### Sampling and analysis

In both studies, feed was withdrawn 4 h prior to sacrifice. All mice were euthanized by decapitation under anesthesia with diethyl ether. Blood was collected into micro tubes (Serum Z, Sarstedt, Nümbrecht, Germany) to gain serum. Serum concentrations of ionized calcium and inorganic phosphate were measured spectrophotometrically using Fluitest® CA CPC and Fluitest® PHOS kits (Analyticon Biotechnologies AG, Lichtenfels, Germany). Serum PTH was analyzed using the mouse PTH 1-84 ELISA Kit (#60-2305) and serum iFGF23 was quantified by using the mouse/rat FGF-23 (intact) ELISA Kit (#60-6800) both purchased from Immunotopics, Inc. (San Clemente, CA, USA). To identify whether high dietary calcium concentrations may induce vascular calcification, aortic root sections were prepared and quantified via von Kossa staining (Schmidt et al., 2012). The area of calcification was related to the aortic valve tissue.

### Statistical analyses

All data were tested for normal distribution by the Shapiro-Wilk test and for homoscedasticity by the Levene test. In the first study, a three-way analysis of variance (ANOVA) was conducted with the classification factors genotype (WT vs. VDR KO), age (6 vs. 13 months), sex (female vs. male), and their interactions (SPSS version 22.0; IBM, Armonk, USA). Each group comprised 5 to 6 mice. In the second study, means of 5 groups (*n* = 5–6) were compared by one-way ANOVA followed by Tukey's test in case of variances homogeneity, or by Welch's ANOVA followed by Games-Howell test in case of variances heterogeneity. Means were considered significantly different at *P* < 0.05.

## Results and discussion

**Figure 1:**
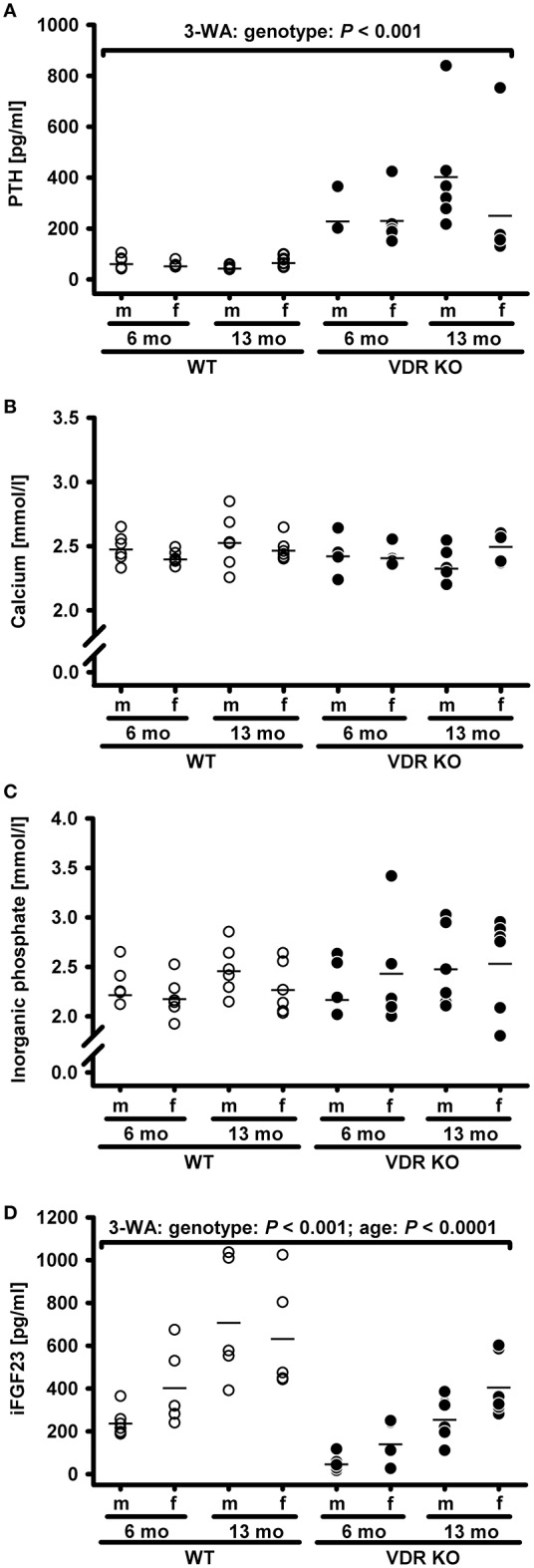
**Serum concentrations of (A)** parathyroid hormone (PTH), **(B)** calcium, **(C)** inorganic phosphate, and **(D)** intact fibroblast growth factor 23 (iFGF23) in male (m) and female (f) wild type (WT) and vitamin D receptor knockout (VDR KO) mice aged 6 or 13 months (mo). Mice were fed a rescue diet with 2% calcium and 1.25% phosphorus. Shown are single data (◦) and means (−) (*n* = 5–6). Data were analyzed by three-way analysis of variance (3-WA) with the classification factors genotype, age, sex, and their interactions.

Vitamin D receptor (VDR) knockout (KO) mice are an important model to unravel VDR-dependent pathways and to study the role of vitamin D in prevention of diseases, e. g. cardiovascular and metabolic diseases. These mice are usually fed a high calcium, high phosphorus diet, termed RD, allegedly to normalize their mineral and PTH status. To elucidate the necessity and efficiency of this diet to normalize the mineral status and the serum PTH concentrations, we first characterized the individual response of VDR KO mice to feeding the classical RD with 2% calcium and 1.25% phosphorus. We found that the mean concentration of circulating serum PTH in VDR KO was significantly higher than that of WT mice placed on the same diet (Figure 1A). More importantly, the PTH serum concentrations differed strongly in the VDR KO mouse group, ranging from quite normal to extremely high PTH levels when compared with WT mice. An insufficient supply of calcium is the major trigger for increased PTH synthesis and secretion from the parathyroid gland. VDR KO mice are characterized by a strongly reduced intestinal transcelluar Ca^2+^ transport, because the apical calcium channel transient receptor potential vanilloid type 6, the calcium binding protein calbindin-D_9k_ and the basolateral plasma membrane Ca^2+^ ATPase are normally under the control of vitamin D (reviewed in Wasserman, 2005). However, besides the transcellular pathway, calcium can also enter the body by a paracellular transport. The paracellular Ca^2+^ transport is independent of vitamin D and is suggested to be controlled by tight junction structures, such as claudin proteins (Amasheh et al., 2005; Inai et al., 2005; Fujita et al., 2006). We speculate that the VDR KO mice could differ in their individual capacity to compensate low calcium levels via the paracellular pathway, which may explain the large variability in the response of VDR KO mice to the ND and RD diets. However, sex and age had no obvious impact on the PTH response and can be excluded as important factors that modulate PTH response in mice. No significant impact of the genotype, sex and age were found on the concentrations of circulating calcium and inorganic phosphate (Figures 1B,C). In addition to PTH, the second hormone iFGF23 concentrations also differed between the VDR KO and the WT mice. The serum concentration of iFGF23 was on average lower in the VDR KO mice than in the WT mice (Figure 1D), a phenomenon which has already been described by Shimada et al. (2005). Despite this well-known impact of VDR deficiency on iFGF23, we also found a significant effect of age on iFGF23, in terms of higher iFGF23 concentrations in the 13-month-old compared to the 6-month-old mice. However, an interaction between genotype and age was not observed.

**Figure 2:**
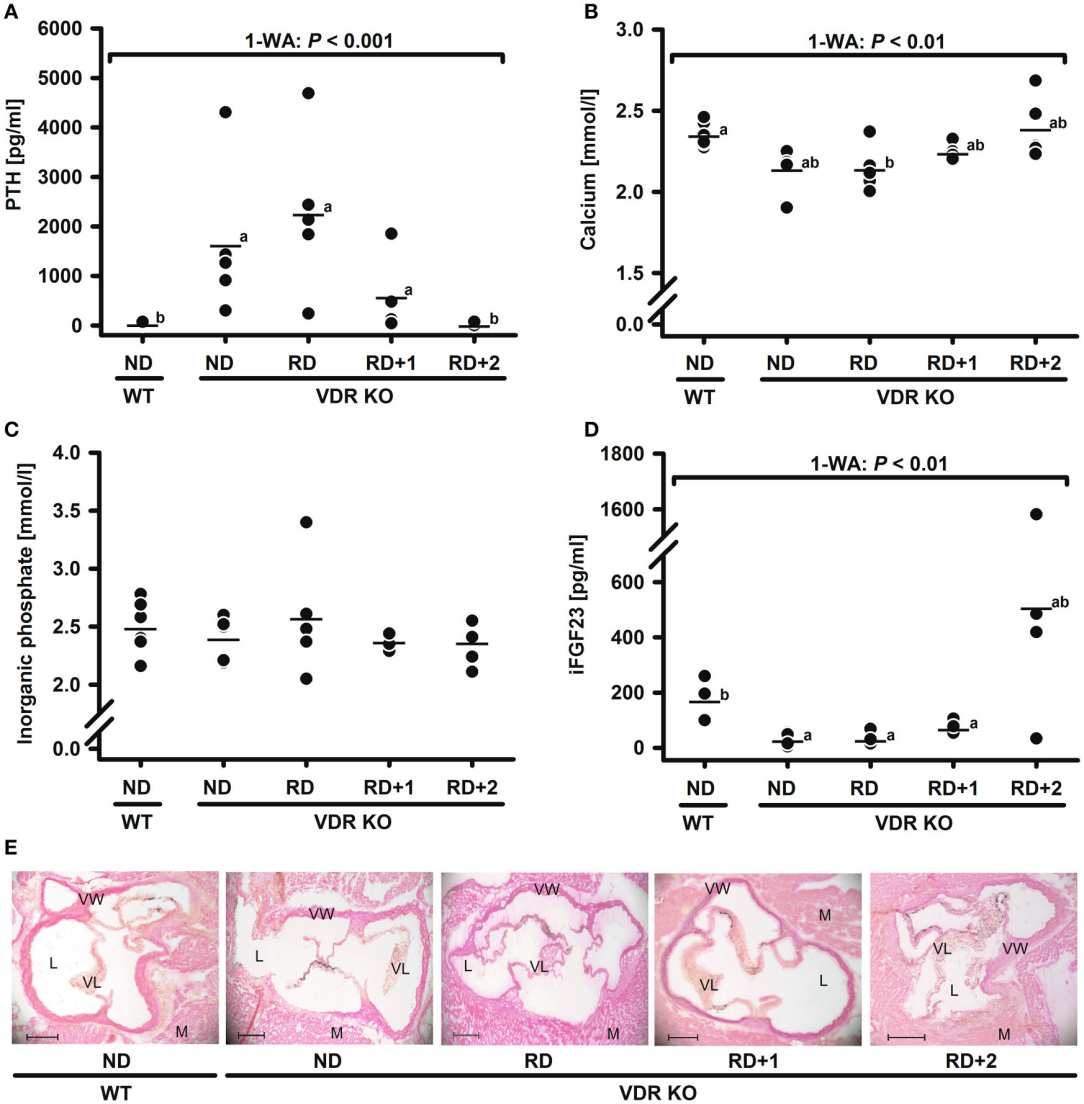
**Serum concentrations of (A)** parathyroid hormone (PTH), **(B)** calcium, **(C)** inorganic phosphate, **(D)** intact fibroblast growth factor 23 (iFGF23), and **(E)** representative images of the aortic root calcification (black spots) in 4-month-old male wild type (WT) and vitamin D receptor knockout (VDR KO) mice after feeding diets differing in minerals for 8 weeks. Mice were fed either a normal diet (ND) added with 0.3% calcium and 0.156% phosphorus, a rescue diet (RD) added with 2% calcium and 1.25% phosphorus, a RD with additionally added 1% calcium (RD+1) or 2% calcium (RD+2). Shown are single data (◦) and means (−) (*n* = 4–6). Data were analyzed by one-way analysis of variance (1-WA). Means not sharing a common letter are significantly different (*P* < 0.05). Aortic root sections: 7 μm, von Kossa stained, images in 5x magnification, scale bars indicate 200 μm; anatomic structures: (L), lumen; (M), myocard; (VL), valve leaflet; (VW), vessel wall.

To define the diet composition, in particular the dietary calcium content, required to normalize serum calcium and to prevent secondary hyperparathyroidism in all VDR KO mice, a subsequent study was conducted. The major finding of this study was that VDR KO mice fed the classical RD had on average serum concentrations of PTH and calcium that were not significantly different from those of VDR KO mice fed the ND (Figures 2A,B). An addition of 1% calcium to the RD was not able to significantly reduce the serum PTH concentrations of the VDR KO mice, although the variation of serum PTH levels became smaller (Figure 2A). Only the VDR KO mouse group fed the RD + 2% calcium reached PTH levels that were not different from those of the WT mice and that showed only minimal variations among the group. PTH is suggested to have wide-ranging physiological effects beyond maintaining calcium and phosphate homeostasis. Analyses from rodent and human tissues revealed that PTH receptors (Pth1 and Pth2) are not only found in the classical target tissues kidney and bone, but are also expressed in brain, pancreas, heart, lung, and in cells of the cardiovascular system (Ureña et al., 1993; Usdin et al., 1995). Data indicated that PTH excess is linked to endothelial dysfunction (Almqvist et al., 2011) and arterial hypertension (Heyliger et al., 2009). Moreover, hyperparathyroidism has been shown to strongly influence a series of genes in the human adipose tissue that are involved in inflammatory response, and in fatty acid and steroid metabolism (Christensen et al., 2011). In addition, PTH appears to be able to stimulate DNA synthesis in enterocytes and subsequently stimulates the intestinal cell proliferation via phosphorylation and activation of the extracellular signal-regulated mitogen-activated protein kinase (MAPK) isoforms ERK1 and ERK2 (Gentili et al., 2001). Those PTH induced effects have to be considered in studies that address the physiological impact of VDR in the VDR KO mouse model.

Analyses from the second study further demonstrated that there was no dietary impact on the serum concentration of inorganic phosphate (Figure 2C) and no impact on aortic root calcification. The relative calcification area was in all groups below 2% of the aortic valve tissue (data not shown). Representative images of stained aortic root sections are shown in Figure 2E. However, the VDR KO mouse group that was fed the RD + 2% calcium, and that was characterized by PTH concentrations comparable to those of the WT mice group, exhibited strongly varying serum concentrations of iFGF23, ranging between 32.1 and 1581 pg/mL (Figure 2D). Similar to PTH, high serum concentrations of iFGF23 may also impact several pathways. FGF23 is synthesized and secreted by osteocytes and acts on the renal proximal tubule, where it lowers the expression of sodium-phosphate cotransporters and increases the excretion of phosphate (Jüppner, 2011). Recent evidence, reviewed by Kanbay et al. (2017) and Haffner and Leifheit-Nestler (2017) pointed out that FGF23 is also involved in the regulation of iron metabolism, inflammation and insulin, and high levels of FGF23 are linked to left ventricular hypertrophy (Haffner and Leifheit-Nestler, 2017; Kanbay et al., 2017). There is also increasing evidence that FGF23 can influence hippocampal neurons (Liu et al., 2011). These FGF23 functions may also limit the conclusions drawn from the VDR KO mice.

In conclusion, the classical RD provides no obvious advantages in normalizing PTH levels when compared with a normal mouse diet. VDR KO mice showed a great variation in their PTH response to the RD, ranging from normal to excessive PTH values. We assume that the high PTH variability of VDR KO mice in the response to a RD probably explains the disparity between studies in the effectiveness of the RD to normalize serum PTH. High dietary calcium concentrations of 4% were efficient in lowering the serum PTH concentration of all VDR KO mice, but were associated by a marked increase in serum iFGF23 concentration in some mice. PTH and FGF23 are two hormones with wide-ranging physiological effects far beyond maintaining calcium and phosphate homeostasis. Thus, data obtained from VDR KO mice studies cannot be attributed to the absent VDR alone. In order to at least ensure a certain degree of homogeneity concerning PTH and iFGF23 within a VDR KO mouse group, the selection of animals with a comparable hormone status is recommended.

## Author contributors

CB, HK, and GS conceived and designed the experiment. DR performed the experiment. CB, DR, and SG analyzed the data. CB, GS, and SG wrote the manuscript.

## Funding

This work was partly funded by a grant from the German Federal Ministry of Education and Research [Grant No. 01EA1411C].

### Conflict of interest statement

The authors declare that the research was conducted in the absence of any commercial or financial relationships that could be construed as a potential conflict of interest. The reviewer MT and handling Editor declared their shared affiliation, and the handling Editor states that the process nevertheless met the standards of a fair and objective review.

## Abbreviations

FGF23: fibroblast growth factor 23
KO: knockout
ND: normal diet
PTH: parathyroid hormone
RD: rescue diet
VDR: vitamin D receptor
WT: wild type.
